# Intervention Integrity in Mindfulness-Based Research

**DOI:** 10.1007/s12671-018-0886-3

**Published:** 2018-01-24

**Authors:** Rebecca S. Crane, Frederick M. Hecht

**Affiliations:** 1 Centre for Mindfulness Research and Practice, School of Psychology, Bangor University, Bangor, LL57 2AS UK; 2 Osher Center for Integrative Medicine, University of California, San Francisco, USA

## Abstract

Assessing program or intervention fidelity/integrity is an important methodological consideration in clinical and educational research. These critical variables influence the degree to which outcomes can be attributed to the program and the success of the transition from research to practice and back again. Research in the Mindfulness-Based Program (MBP) field has been expanding rapidly over the last 20 years, but little attention has been given to how to assess intervention integrity within research and practice settings. The proliferation of different program forms, inconsistency in adhering to published curriculum guides, and variability of training levels and competency of trial teachers all pose grave risks to the sustainable development of the science of MBPs going forward. Three tools for assessing intervention integrity in the MBP field have been developed and researched to assess adherence and/or teaching competence: the Mindfulness-Based Cognitive Therapy-Adherence Scale (MBCT-AS), the Mindfulness-Based Relapse Prevention-Adherence and Competence Scale (MBRP-AC), and the Mindfulness-Based Interventions: Teaching Assessment Criteria (MBI:TAC). Further research is needed on these tools to better define their inter-rater reliability and their ability to measure elements of teaching competence that are important for participant outcomes. Research going forward needs to include systematic and consistent methods for demonstrating and verifying that the MBP was delivered as intended, both to ensure the rigor of individual studies and to enable different studies of the same MBP to be fairly and validly compared with each other. The critical variable of the teaching also needs direct investigation in future research. We recommend the use of the “Template for Intervention Description and Replication” (TIDieR) guidelines for addressing and reporting on intervention integrity during the various phases of the conduct of research and provide specific suggestions about how to implement these guidelines when reporting studies of mindfulness-based programs.

## Introduction

The scientific investigation of Mindfulness-Based Programs (MBPs) has progressed rapidly in the last 20 years. A frequently employed and effective way to demonstrate this expansion is by citing the number of peer-reviewed publications with “mindfulness” in the title. In 1984, there were two papers, whereas in 2016, there were 856 such papers (based on a search of the Web of Science database on 26 June 2017). There have been voices of caution within the field regarding this proliferation of research, the potential for gaps in the methodical development of the science, and calls for greater levels of rigor and strategic thought in research developments going forward (Dimidjian and Segal 2015; Van Dam et al. 2017).

A central issue in the study of MBPs, which we believe needs to be better addressed for the field to advance, is the issue of intervention integrity. Intervention integrity is defined as ensuring that the intervention was delivered as intended (Perepletchikova et al. 2007). Intervention integrity is a delicate and challenging area in many types of non-pharmacological intervention research in which the intervention is delivered by a person. Randomized controlled trials (RCTs) were initially designed to investigate drugs, for which it is straightforward to standardize dose and ingredients. It is difficult to standardize and operationalize the behavior of the person delivering the program. MBPs are complex interventions with multiple elements to be accounted for during implementation (Craig et al. 2006). One key emphasis within MBP teacher training and program delivery is the importance of embodied communication of mindfulness by the teacher, which draws on the teacher’s personal practice of mindfulness. This strong reliance on a certain sort of inner work within the teacher to enable effective teaching practice presents challenges to researchers in their work of unpacking and analyzing the critical ingredients of MBPs, and ensuring that the intervention was delivered as intended.

One approach to ensuring intervention integrity in the context of complex interventions, including some MBPs, has been the development of detailed intervention manuals and assessment of whether the manual was adhered to. This approach has been encouraged by the National Center for Complementary and Integrative Health (NCCIH) (2017), which funds a substantial amount of the MBP research in the USA, and it has been applied in different trials of mindfulness interventions (Daubenmier et al. 2016; Mackenzie et al. 2006; Vieten and Astin 2008). Simply assessing whether manualized curriculum topics and pacing were adhered to, however, may overlook some of the most important elements of intervention delivery. As one example, Daubenmier et al. conducted a clinical trial testing whether adding mindfulness components (mindful eating and many elements from MBSR) to a diet and exercise intervention was more effective than diet and exercise alone for weight loss maintenance for people with obesity (Daubenmier et al. 2016). At 18 months, there were statistically significant differences in weight loss between participant groups within the mindfulness arm, depending on who led the groups. Weight loss at 18 months was correlated with participant ratings of how helpful the teacher was 1 year earlier. Although there were only three teachers to compare, the differences did not appear to be explained by experience (all teachers had substantial experience), nor by adherence to the intervention manual. In fact, the teacher with the weakest outcomes appeared to be most adherent to the timing elements specified in the manual. Although our data cannot establish this with any certainty, our experience suggested that the effort to adhere closely to delivering elements specified in the intervention manual might have detracted from elements important to intervention potency, such as the ability to convey course themes through interactive inquiry, and the capacity to embody the practice of mindfulness. This implies that manualization alone is not the answer to assuring intervention integrity in MBPs, and underlines the potential importance of methods to assess the components of teacher competence that matter most for intervention potency. In another example, Huijbers et al. (2017) analyzed the links between MBP teacher competence and participant outcome. While no significant link in this particular study was found, there were differences between teachers. Preliminary evidence in the MBP field indicates that teacher factors could influence medium significant effects in an adequately powered study (Prowse et al. 2015). Taken together, these suggest that this issue of teacher effects is an area ripe for investigation.

Intervention integrity is a critical issue for the field going forward because the systematic process of building the evidence base relies on the integrity of each individual research study, and the comparability of research outcomes from different studies on the same programs relies on whether they were delivered in similar ways. The intervention delivery is a critical variable within the research process, and if it cannot be verified that it was delivered as intended, it is difficult to meaningfully interpret the outcomes of the study (Sharpless and Barber 2009). Meaningful fidelity checks may enable nuanced analysis of the potential reasons for particular study outcomes. For example, it becomes possible to analyze whether outcomes may have been influenced by differing levels and sorts of teacher training, adherence to good practice norms, or whether specific domains of teacher competence are important for particular outcomes. All these issues can feed into the development of future research questions (Herschell 2010).

No single trial is enough to give definitive results. It is through each trial contributing to a larger corpus of knowledge synthesized in systematic reviews and meta-analyses, that we can begin to see patterns based on overlaps and differences in populations, comparator conditions, outcomes, and characteristics of the program, itself. It therefore becomes a critical issue that each contributing trial is of the highest quality possible.

In the current wave of expanded interest in MBPs, there is a proliferation of new program forms. This is part of a creative response to the need to adapt programs to new contexts and the populations but does create challenges in building an evidence base for MBPs. There can be an assumption that research results derived from one MBP form can be interpreted in light of results derived from another. Factors that can confound this include deviation from a published curriculum while still labelling it with the original title, and variations in the quality of the teaching itself. If an MBP does not adhere to existing curriculum protocols, it is an important matter of accuracy, ethics, and careful science to ensure that it is given a new title or, deviations and adaptations be carefully documented in the paper.

We summarize the status of understanding on teacher integrity/fidelity issues in the MBP field, underline the importance of assessing intervention integrity for the forward development of the science, and offer guidance on addressing it within the various phases of conducting research. We discuss a number of related areas—the level of adherence to the program being researched, the level of competence of the teacher(s) delivering the program, the teacher’s adherence to norms of good practice, and their training and experience prior to teaching within a research trial. The aim is to lay out good practice guidance for researchers of MBPs during the design, conduct, and reporting phases of research on the issues of integrity of the MBP within their research. We use the term MBP in the way it is defined by Crane et al. (2017). The term “intervention” is used at points to emphasize linkage to the broader literature on intervention integrity. However, in the context of the mindfulness field, the term “program” is preferred because it speaks to the wider use of MBPs in a range of contexts beyond health care.

### Status of Understanding on Teaching and Program Integrity in the MBP Field

The concept of intervention integrity or fidelity arises out of research on educational and psychotherapeutic programs. Several conceptual models of treatment integrity have been proposed (Sanetti and Kratochwill 2009). A commonly used conceptual model of treatment integrity in the psychotherapy field uses three dimensions: adherence, differentiation, and competence (Borrelli 2011; Weck et al. 2011). Adherence and differentiation are closely related content aspects of integrity: how frequently the teacher/therapist delivers prescribed intervention procedures (adherence) and omits proscribed elements (differentiation), and to what degree these procedures are employed to ensure intervention “purity.” Competence is the skill level of the therapist/teacher in delivering the intervention. While adherence, differentiation, and competence are related, they do not presuppose each other. In particular, delivering an intervention with adherence and differentiation does not necessarily mean the intervention has been delivered competently.

**Fig. 1:**
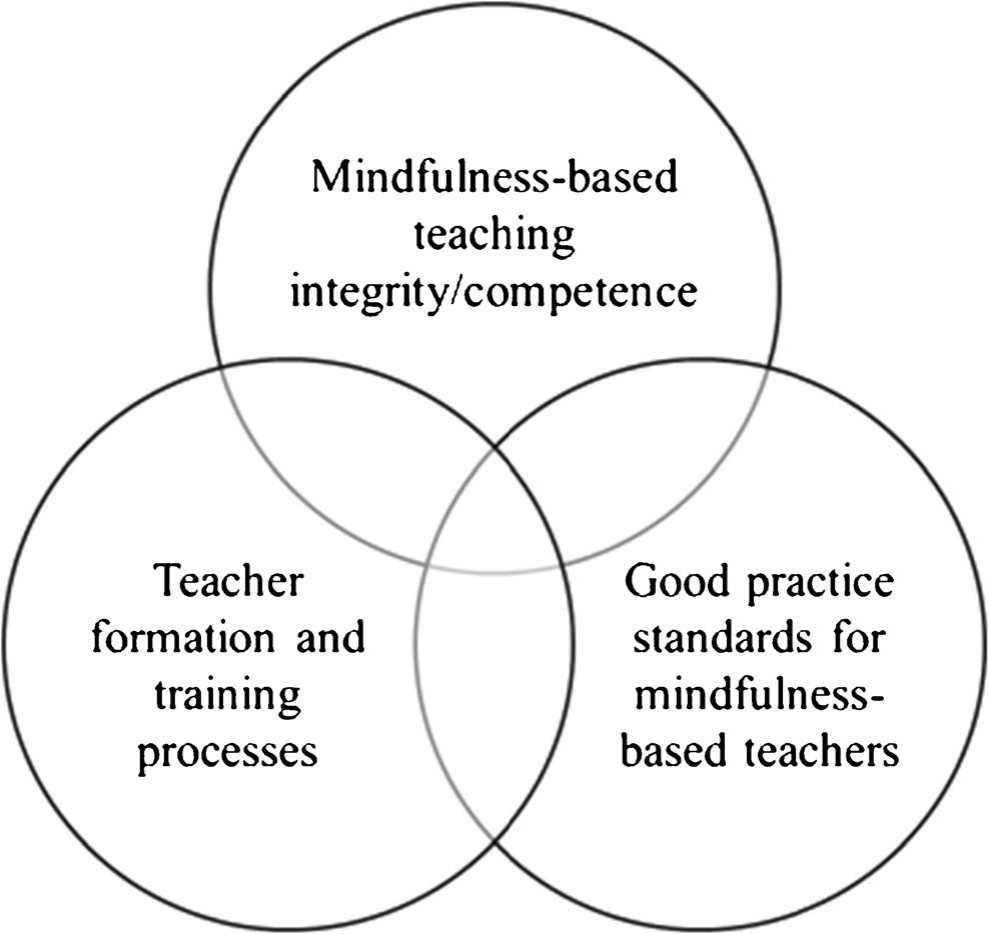
Three interconnected aspects of quality and integrity in teaching mindfulness-based courses (from Crane et al. 2012)

Intervention integrity, particularly the dimension of teacher competence, links to three interconnected areas: standards/guidelines for good practice for teachers, models for training teachers, and methods of understanding and assessing program integrity (Crane et al. 2012) (see Fig. 1).

### Good Practice Guidelines (GPGs)

In recent years in the MBP field, there have been concerted efforts to develop and communicate agreed upon norms for good practice for both teachers and trainers of teachers. Some have arisen in national and regional collaborations of trainers (UK Network for Mindfulness-Based Teacher Training Organisations 2016), of teachers (European Association of Mindfulness based Approaches (EAMBA 2017), and in other examples, have been coordinated by a training organization in collaboration with international colleagues (Center for Mindfulness in Medicine, Health Care and Society, University of Massachusetts Medical School 2014; Segal et al. 2016). There are differences in detail, but much alignment on general principles within these guidelines. They all outline minimum teacher training levels, stipulate that the teacher engages in a personal daily mindfulness practice combined with periodic intensive residential mindfulness practice opportunities, a commitment to on-going development through further training, keeping up with the evidence base, supervision, linkage with colleagues, and adherence to an ethical code of conduct. There is currently no direct empirical support for particular ingredients within GPGs, and there is ample room for scientific study of the effects of (for example) regular supervision on teaching practice, and attendance on residential mindfulness practice intensives on the teacher’s capacity to embody and communicate mindfulness. The GPGs have though emerged through a rigorous process of consensus building by highly experienced MBP trainers, and are based on evidence in related fields, and on understanding of MBP pedagogy.

### Teacher Training Models

There is considerable practice-based evidence and understanding on this theme, which has been disseminated both informally and via journal articles (e.g., Crane et al. 2010; Dobkin and Hassed 2016; Marx et al. 2015). Similar to the GPG issue above, there is little empirical analysis of the effects of teacher training models on building competence and on participant outcomes. There is the beginning of research activity in this area, however. For example, van Aalderen et al. (2014) conducted a triangulated qualitative analysis of how the MBCT teacher-participant relationship impacts participants. This study found that teacher embodiment of mindfulness, empowerment of participants, teacher non-reactivity, and group support were important factors in the teaching process. Ruijgrok-Lupton et al. (2017) conducted an investigation of the impact of teacher training on participant outcomes. They found that participants’ gains after taking an MBSR program were correlated with teacher training and experience—gains in wellbeing and reductions in perceived stress were significantly larger for the participant cohort taught by teachers who had completed an additional year of mindfulness-based teacher training that involved assessment of teaching competence. Kuyken et al. (2017) have integrated investigation of the comparative effects of lighter and more substantial teacher training on outcomes of school children into the protocol for a trial on mindfulness in schools.

### Methods of Assessing Intervention Integrity

The development and validation of assessment methods for MBP competence is at an early stage in the field (see Table 1 for a summary of the methods currently available). Currently, the MBI:TAC (Crane et al. 2013; Crane et al. 2016) is the most commonly used tool within the field in both training and research contexts. It focuses primarily on assessing teaching competence within the context of MBSR and MBCT, though an addendum has been developed for the Mindfulness in Schools program (Mindfulness in School Project 2017), and work is underway to develop an addendum for MBP teaching in workplace contexts. The MBI:TAC was a collaborative development led by Bangor University with Exeter and Oxford University mindfulness centers. The primary aim for the initial development was to create a reliable and valid system for assessing MBSR/MBCT teacher trainee’s teaching practice within post-graduate training programs. It describes six domains within the teaching process: coverage, pacing, and organization of session curriculum; relational skills; embodiment of mindfulness; guiding mindfulness practices; conveying course themes through interactive and didactic teaching; and holding the group-learning environment. Within each domain, it identifies key features that unpack the elements within that domain, and levels of competence (incompetent, beginner, advanced beginner, competent, proficient, and advanced). The person performing an assessment using the MBI:TAC needs to be an experienced teacher of MBPs, experienced in teaching the particular MBP that is the subject of the assessment, and trained to use the tool reliably. S/he gathers their observational data via experiential participation in a piece of teaching (either in person or through audio-visual recordings), and then systematically applies the criteria to make an assessment point within each domain.

**Table 1:** Tools for assessing MBP intervention integrity

Tool	Target MBP	Which aspects of intervention integrity it assesses	Publications	Focus of research
Mindfulness-Based Cognitive Therapy-Adherence scale (MBCT-AS)	MBCT	Adherence	Segal et al. (2002)	Initial evaluation of psychometric properties
			(Prowse et al. 2015)	Research on the tool embedded within an MBCT trial
Mindfulness-Based Relapse Prevention Adherence and Competence Scale (MBRP-AC)	MBRP	Adherence, competence	(Chawla et al. 2010)	Psychometric properties
Mindfulness-Based Interventions: Teaching Assessment Criteria (MBI:TAC)	MBSR, MBCT, Adaptation made for Mindfulness in Schools program	Adherence, differentiation, competence	(Crane et al. 2013)	Initial evaluation of psychometric properties
			(Huijbers et al. 2017)	Analysis of links between participant outcome and teacher competence as assessed by MBI:TAC

Preliminary research on the psychometric properties of the tool demonstrated good inter-rater reliability (intra-class correlation coefficient; *r* = .81, *p* < .01). The evaluations of validity that were possible at this early stage in the tool’s development were encouraging, but there are important limitations of this initial validation work. Although 43 different teachers were rated, only two assessments were used for assessing reliability, which limits the precision of the estimates of inter-rater reliability. In addition, raters were aware of the level of experience of the teachers they were rating, which may have influenced ratings. Further research in a range of contexts is needed to clarify the MBI:TAC’s reliability and validity. The only study so far to use the MBI:TAC to investigate links between teacher competence and participant outcome, did not find significant effects on mediators and outcome variables in MBCT for recurrent depression (Huijbers et al. 2017). Further work is required to systematically investigate these important issues.

The MBI:TAC is a set of criteria rather than a measure of teacher competence. As such, it requires the user of the tool to have training to ensure that the criteria are being applied consistently—one person’s idea of “competent” might be another person’s idea of “advanced.” It is therefore important to ensure that the use of the tool does not rely on the ideas and interpretations of the user (which are inevitably biased by cultural, educational, and personal conditioning) but is based on training towards centralized norms of what a competent teaching of a sitting meditation in week 5 of an MBSR looks like (for example). Assessors therefore need to engage in a training process to build their reliability in using the tool and alignment of their assessments to central benchmarked assessments.

The MBI:TAC does seem to have face validity in that it is being implemented in MBP training centers worldwide both as an assessment tool and as a tool to support reflection on skills development (Evans et al. 2014; Marx et al. 2015). It offers to trainers and trainees a useful orienting map of the territory of the competencies being developed.

There are other tools that have been developed to assess MBP integrity/fidelity. The MBCT-Adherence Scale (MBCT-AS) is a 17-item scale designed to assess the presence/absence of MBCT curriculum elements and principles (Segal et al. 2002). Individual items are rated as “no evidence”, “slight evidence” or “definite evidence”. Inter-rater reliability was tested during the original MBCT research trials (Ma and Teasdale 2004; Teasdale et al. 2000), and with intra-class correlation coefficients (ICC) ranges from.59 for the cognitive therapy subscale, .97 for the mindfulness subscale and.82 for global ratings. A subsequent study employing the MBCT-AS (Prowse et al. 2015) demonstrated the value of implementing fidelity assessment within delivery of an RCT—fidelity assessment “proved critical in diagnosing program weaknesses and identifying program strengths to support improved treatment delivery” (p. 1407). There are several limitations of this scale at present to assess MBP integrity/fidelity. First, the instrument focuses mainly on adherence to intervention content rather than teacher competence; second, the scale is primarily intended for use with MBCT and, to our knowledge, has not been adapted for use with other MBPs; third, the initial assessment of inter-rater reliability was done with only 3 raters rating 16 audiotapes. This is a small number for assessing inter-rater reliability (Saito et al. 2006); hence, the inter-rater reliability is not fully established. Finally, like other instruments, the relationship between items on this instrument and participant outcomes has not been fully assessed.

The Mindfulness-Based Relapse Prevention Adherence and Competence Scale (MBRP-AC) (Chawla et al. 2010) is a measure of the intervention integrity of MBRP that was developed in the context of a randomized controlled trial. A strength of this scale is that it includes both an adherence section (level of fidelity to individual components of MBRP and delivery of key concepts), and a competence section (ratings of teaching style and approach). Inter-rater reliability was generally good, and ratings on the adherence section were positively related to changes in mindfulness over the duration of the program. Like the MBCT-AS, it was designed for a particular intervention, and adaptation may be needed to apply it to other MBPs, although the competence domains (inquiry, attitude/modeling of mindfulness, use of key questions, and clarifying expectations) may readily transfer to other MBPs. In assessing inter-rater reliability, a substantial number of sessions were assessed (44) but only by 2 raters, limiting the precision of the estimates of inter-rater reliability. In addition, some of the ICC results on scale items were just above the threshold of 0.5, which has been considered the lower range of moderate reliability (Koo and Li 2016): of 13 items, 4 had ICCs between 0.5 and 0.6. If 95% confidence intervals had been provided, as would be ideal for evaluating the precision of the ICC estimate, the lower bound would almost certainly have been below 0.5, an ICC that is considered to show poor inter-rater reliability.

### Integrating Assessment of Intervention Integrity into the Phases of Research

The CONSORT (Consolidated Standards of Reporting Trials) guidelines provide an important set of good practices for reporting clinical trials (Schulz et al. 2010). These include standard elements for authors to describe when preparing reports of trial findings, facilitating their complete and transparent reporting, and aiding their critical appraisal and interpretation. The element most applicable to the issue of intervention fidelity is item 5, which involves describing the: “interventions for each group with sufficient detail to allow replication, including how and when they were actually administered.” The CONSORT guidelines also include an extension for reporting non-pharmacological intervention trials that is helpful in addressing the additional issues involved in reporting MBPs (Boutron et al. 2008). Item 4 in this extension outlines additional elements for non-pharmacologic trial intervention reporting, includes reporting details of the intervention components, how the interventions were standardized, and how adherence to the protocol implementation was assessed.

Another recent set of recommendations, which expands item 5 within the CONSORT guidelines by providing detailed guidance on how to report intervention integrity issues, is the Template for Intervention Description and Replication (TIDieR) guidelines (Hoffmann et al. 2014). These provide a much more detailed set of recommendations for how to report interventions so that adequate information is provided to allow replication. We believe the TIDieR guidelines provide an important roadmap for improving reporting on the intervention component of MBP trials in general, and how intervention fidelity was addressed. Such guidelines are important not only for researchers, but for all of us who read the research literature to inform our practice. In the following sections, we describe how we suggest researchers performing trials of MBPs might best apply the TIDieR guidelines when planning and conducting MBP trials, and how these steps are reported when publishing the trial. Table 2 summarizes these TIDieR guidelines and their relevance to the MBP research context.

**Table 2:** Items included in the Template for Intervention Description and Replication (TIDieR) checklist: information to include when describing an intervention, with additional guidance (in italics) on applications to MBP research

Item number	Item
Brief name	
1.	Provide the name or a phrase that describes the intervention and *reference to the most recent curriculum guide—*i.e., *MBSR* (Santorelli et al. 2017*)*
Why	
2.	Describe any rationale, theory, or goal of the elements essential to the intervention. *In addition to referencing published literature on this issue, theoretical rationales are needed for any adaptations, or tailoring to a particular population or context.*
What	
3.	Materials: Describe any physical or informational materials used in the intervention, including those provided to participants or used in intervention delivery or in training of intervention providers. Provide information on where the materials can be accessed (such as online appendix, URL). *For example, written course materials and guided mindfulness meditation practices.*
4.	Procedures: Describe each of the procedures, activities, and/or processes used in the intervention. *If using a published MBP curriculum guide this is not needed—only include descriptions of adaptations. Detail in full if delivering a new MBP.*
Whom provided	
5.	For each category of intervention provider, describe their expertise, background, and any specific training given. *Describe (1) what MBP teacher training has been undertaken by trial teachers, (2) how they adhere to ongoing MBP Good Practice Guidelines such as on-going practice, and (3) measures of teacher competence that were used to select trial teachers*
How	
6.	Describe the modes of delivery (such as face to face or by some other mechanism, such as internet or telephone) of the intervention and whether it was provided individually or in a group. *If following a standard MBP curriculum guide this is not required—only detail deviations/adaptations from standard protocols, or if a new curriculum, detail in full, including delivery method* (i.e., *in person teacher-led group sessions; digital delivery, etc.*).
Where	
7.	Describe the type(s) of location(s) where the intervention occurred, including any necessary infrastructure or relevant features.
When and How Much	
8.	Describe the number of times the intervention was delivered and over what period of time including the number of sessions, their schedule, and their duration, intensity, or dose. *If following a standard MBP curriculum guide this is not required—only detail deviations/adaptations from standard protocols, or give full details of new MBPs.*
Tailoring	
9.	If the intervention was planned to be personalized, titrated, or adapted, then describe what, why, when, and how. *Describe how individual needs/vulnerabilities of MBP group participants were handled by the trial teacher(s), and whether any steps such as individualized additional meetings with the teacher were used to address issues that varied by participant.*
Modifications	
10.	If the intervention was modified during the course of the study, describe the changes (what, why, when, and how).
How well	
11.	Planned: If intervention adherence or fidelity was assessed, describe how and by whom, and if any strategies were used to maintain or improve fidelity, describe them. *Describe whether an MBP fidelity tool was used to assess intervention delivery* via *reviews of recorded sessions were employed, by whom and how. Describe the rationales for the choices made.*
12.	Actual: If intervention adherence or fidelity was assessed, describe the extent to which the intervention was delivered as planned. *Detail the assessed level of MBP teaching competence, adherence and differentiation in the results section of the paper.*

Adapted from Table 1 in Hoffmann et al. (2014)

*Item 1* of the TIDieR guidelines is to “provide the name or a phrase that describes the intervention.” For planning and reporting MBPs, this means addressing a critical first question: defining which MBP is being studied. If an existing MBP is being employed it is important to ensure that the delivered curriculum maps exactly onto the manual or curriculum guide for this MBP (Hoffmann et al. 2014). For MBSR curriculum guide see (Santorelli et al. 2017); for MBCT see (Segal et al. 2013) and for other MBPs specific guidelines are available. If the adaptations are significant, the MBP needs a new name. A challenging question is how much adaptation can take place before an MBP needs a new title (Dobkin et al. 2013). Crane et al. (2017) provide a meta-perspective on this question in the context of all MBPs by defining the essential and variant ingredients and qualities of any program that is *based* on mindfulness. Researchers then need to narrow these questions down to the specifics of the program under consideration. There are no definitive answers but there are some important elements, including (a) the dosage (i.e., if calling a program MBSR it needs to include a minimum of 31 hours of direct instruction plus assignment of 45 mins per day of formal home practice); (b) delivery and sequencing of the core meditation practices (i.e., in MBCT these are the body scan, mindful movement, sitting meditation, and the 3-min breathing space, each taught over particular durations, in particular ways at particular time points within the program); and (c) the core themes of each session as laid out within the curriculum guide. An acceptable level of adaptation (while retaining the particular MBP title), might therefore be adjusting the psychoeducational material to a particular population (which in turn is informed by understanding of the mechanisms by which vulnerability is created and perpetuated in this population); or by adjusting the delivery format (but not the overall dosage) to suit the constraints of a particular context.

*Item 2* in the TIDieR guidelines is to describe the rationale or theory of the intervention elements. For MBPs, this means defining and reporting why the particular MBP was selected for study, and the theoretical model by which it is hypothesized to be effective in the study context. If program adaptations are made, investigators should make sure they have a clear rationale for the adaptations, which is described in publications. How does the MBP interface with the particular vulnerabilities/life themes of the participants? How do these vulnerabilities present themselves? How are they perpetuated? How does the MBP interface with the context for delivery? See Crane et al. (2017).

*Items 3, 4, 7*, *and 8* of the TIDieR guidelines include describing a set of detailed curriculum-related items that are challenging for MBP’s due to the complexity of most MPBs. Addressing these items will typically require either referencing an existing manual/curriculum guide, together with noting any adaptations, or publishing a new manual/curriculum guide if this represents a new MBP. While these items might be concisely summarized within the methods section in a trial results publication, a new manual/curriculum guide or a lengthy description of adaptations will typically require publication in one of four formats: (1) a separate trial protocol publication in an appropriate journal (for example, a series of on-line journals now publish detailed trial protocols; (2) as an on-line appendix to the article, if the journal provides such an option; (3) as an on-line resource on a website that will serve as a long-term reference (i.e., is not likely to have the URL change or be abandoned); and (4) as a book (e.g., Segal et al. 2013).

TIDieR *item 3* covers describing what informational or physical materials are used in an intervention. For MPBs, this would typically involve describing (and ideally providing examples) of materials such as handouts for participants and guided meditation audio-tracks.

*Item 4* involves describing the procedures and activities used. For MBPs, this will typically involve noting the types of mindfulness practices performed during in-person sessions (e.g., a 15-min body scan at the beginning of the class meeting), or for home practice. Other in-class activities, such as didactic teaching (e.g., stress reactivity and mindfulness), and group exercises should be described, with enough detail to support consistency by multiple teachers within a trial, or to facilitate replication by other investigators. While specifying detail is challenging for elements such as group exercises, outlining issues such as themes that group leaders aim to address can facilitate replication and provide items that are useful in assessing fidelity to intervention curriculum. All the teachers within a trial need to be working to the same curriculum guide.

Clarity is needed within trial teacher training processes regarding how to address adherence. For example, some trials take the line of requiring inclusion of certain poems within certain sessions, and standardization of the audio recordings of meditations given to the participants for home practice. However, another approach is to address adherence by seeing it as adherence to the essence of the process of teaching MBPs. In this case, the teachers are encouraged to work responsively in the moment by selecting poetry that meets emergent themes in the teaching space, by working flexibly with the curriculum to enable responsiveness to a theme that has spontaneously emerged, and by offering participants meditation practice recordings with their own teacher’s voice. The field is tending towards the latter. This level of fluidity is entirely in keeping with the spirit of MBP teaching, but the challenge is to ensure that it continues to flourish within overarching agreed norms of understanding about program fidelity.

*Item 5* of the TIDieR guidelines involves describing who delivered the intervention, and what their background, expertise, and specific training was. This encompasses the critical question of whether the teachers selected for teaching on an MBP trial are at an acceptable level of competence, have trained to acceptable levels, and are adhering to accepted norms of good practice. Good trial governance asks that competence checks are conducted on the teachers in advance of embarking on research trial classes. The requirements for this vary depending on the nature and stage of the research. In this section, we refer to the phases of clinical research, as adapted to behavioral intervention research by (Onken et al. 2014).

*Stage II efficacy* research trial (Onken et al. 2014). For this kind of trial, it is important to choose the best available teachers because the trial is asking a proof of concept question. If the teaching is of a poor quality, it will not be possible to determine whether lack of efficacy was the result of poor teaching or a weakness in the intervention itself. If the teaching is of a high quality, this variable has effectively been eliminated, and the outcomes can be interpreted in the light of other issues. While more research is needed about the best ways to assess teacher competence, there are a couple of options that currently exist. One is to establish certain criteria for the type of training that teachers have received, and the level of experience teaching, and report these in the intervention methods. While this may be useful, as noted earlier, this may not fully establish teacher competence. The second method, which can be combined with the first, is to use an instrument such as the MBI:TAC. If the MBI:TAC is being used to assess competence, we recommend that (for stage II trials) the teaching is at “proficient level” or above.

*Stage III and VI trial* (Onken et al. 2014). For these trials, the core research questions are different. By this phase of the research journey, the MBP has been proven to be of value in a carefully controlled research environment. The next phases of investigation are to ask whether it can stand up to the challenge of being implemented in a real world/community setting. During these phases, a legitimate research question could be: what are the effects of different levels of experience/training/good practice/competence within the trial teachers? These could be manipulated in the trial design, or the natural expression of them captured in the data so that these questions can be analyzed. In this phase of research, the key issues are to accurately assess the level of skill and experience of the teacher. If the MBI:TAC is being used to assess competence, the “advanced beginner” level is at a level that is “fit for practice” in that the participants would come to no harm (although their opportunities for learning might be compromised); competent is the level at which teacher trainees are able to graduate from post-graduate programs in the UK context and is generally recommended as a minimum level for trial teaching. Teaching that is at competent level as assessed by the MBI:TAC is a solid demonstration of good practice, with some areas for development.

TIDieR *item 6* involves describing the mode of delivery of the intervention (i.e., face-to-face, digital, individual or group).

TIDieR *item 7* involves describing where the intervention was conducted, and any infrastructure (e.g., a large, carpeted room) that was needed for the intervention.

*Item 8* involves describing the number of sessions involved in the intervention, length of session, and over what period the intervention was delivered.

*Item 9* involves noting any plans to personalize or adapt the intervention for individual participants. Examples of how this might be applied for MBPs include whether any of the practices are modified for specific participant groups (e.g., the mindful yoga postures could be modified in the following ways for participants with limited mobility), or whether individual attention is available for certain participants (e.g., participants reporting difficulty with the mindfulness practices were offered an option of having a 15-min individual meeting with the mindfulness teacher).

TIDieR *items 11 and 12* (planning for and conducting assessments of intervention fidelity): In studies of MBP’s one of the elements of item 11 in the TIDieR guidelines should typically involve creating a plan to assess intervention fidelity during the trial, as well as plans to ensure that the teachers are supported and adhering to field norms of good practice. In the UK context, this includes regular engagement in Mindfulness Supervision (Evans et al. 2014), and (at least annual) residential, teacher-led mindfulness practice intensives (Peacock et al. 2016; UK Network for Mindfulness-Based Teacher Training Organisations 2016).

Assessing intervention integrity involves having at least some sessions observed or recorded and reviewed to assess the degree to which the intervention is implemented in the way it was intended. It is important to decide what protocol to follow in terms of selection of teaching for integrity checks, and who conducts the checking. These issues need to be carefully addressed in the context of the overall trial and reported in trial publications. Decisions will depend on the overall amount of teaching within the trial, the resources available, and the core purpose of the integrity checks. Is intervention integrity part of the research hypotheses/questions, or are the checks to ensure confidence in answering primary efficacy or effectiveness question? If the former, then there will need to be inter-rater reliability checks on the assessment process itself. If the latter, the fidelity assessment outcomes will be important in enabling the trial to be benchmarked against other trials within the field. Typically, if the check is part of trial governance rather than actually contributing to the trial data, an independent assessor will randomly sample one to two sessions per eight-session course for rating. The outcomes will be reported as part of the trial conduct (TIDieR item 12). The assessor conducting the integrity checks needs to be an experienced MBP teacher in the program that is being researched and trained to use the integrity assessment tool to acceptable levels of reliability.

Research governance requires that the trial protocol is established and ideally published, and the trial registered before embarking on the work on the research. The trial’s approach to intervention integrity, teacher training, and good practice for the teachers need therefore to be addressed and included in the reported protocol. When reporting MBP trials, we recommend that authors use the TIDieR guidelines, with the specific adaptations for MBPs outlined here, as a guide to how to achieve a high-quality section on intervention integrity.

## Conclusions

The main theme that we address is how to integrate teaching integrity questions into the conduct of MBP effectiveness and efficacy trials. We hope this paper offers journal editors and peer reviewers clear guidance which will enable them to offer constructive commentary to authors and will in turn shape practice in this area. It also urges the field to focus future research directly on teaching integrity/fidelity issues. Relative to the overall expansion in research on MBPs, there has been little attention to the way that these effects are created—the curriculum and the teaching process themselves. While current developments offer a foundation for next steps, it is also clear that the methodologies to assess teaching integrity within the MBP field are themselves at an emergent stage and need on-going development and refinement informed by empiricism. As Dimidjian and Segal (2015) pointed out, developing empirical understanding of intervention integrity will be a critical foundation for the rigorous and sustainable development of the science. Research on teaching integrity is also important for the process of implementation (both the research on it and the practice of it). At this point in time, there is little direct evidence to support the length and type of teacher training that is stipulated in current GPGs (though see Ruijgrok-Lupton et al. 2017 for a small-scale exception to this). Indirect evidence on rigorous trials that do report teaching integrity underline that the teachers were working to published norms of training and good practice, which supports the GPGs, but direct investigation of these issues is needed going forward. We recommend that researchers of MBPs use the TIDieR framework and supporting resources for ensuring completeness of reporting of the intervention(s) within their study (Hoffmann et al. 2014).

Ultimately, if a research trial is useful to the world, it will contribute to the emerging evidence base, whether its results are positive or negative. Building empirical understanding is an extraordinary process of interconnected human endeavor, with each researcher contributing one piece in an overall jigsaw of understanding. This collaborative knowledge generation works well if each researcher takes responsibility to do what they say they are doing, to do it well, and then to report it transparently and clearly. We hope that this paper provides clarity on one aspect of “doing it well” within the MBP research process. Current understandings on MBP teaching integrity are themselves preliminary and subject to evolution as evidence builds. They do, however, offer us ground to stand on for now and a platform for future development.

## References

[CR1] Borrelli B (2011). The assessment, monitoring, and enhancement of treatment fidelity in public health clinical trials. Journal of Public Health Dentistry.

[CR2] Boutron I, Moher D, Altman DG, Schulz KF, Ravaud P (2008). Extending the CONSORT statement to randomized trials of nonpharmacologic treatment: Explanation and elaboration. Annals of Internal Medicin.

[CR3] Center for Mindfulness in Medicine, Health Care and Society, University of Massachusetts Medical School. (2014). Mindfulness-based stress reduction (mbsr): Standards of practice. Retrieved from https://www.umassmed.edu/contentassets/24cd221488584125835e2eddce7dbb89/mbsr_standards_of_practice_2014.pdf

[CR4] Chawla N, Collinsa S, Bowena S, Hsua S, Growa J, Douglass A, Marlatt GA (2010). The mindfulness-based relapse prevention adherence and competence scale: Development, interrater reliability, and validity. Psychotherapy Research.

[CR5] Craig, P., Dieppe, P., Macintyre, S., Michie, S. and Nazareth, I. (2006). Developing and evaluating complex interventions: New guidance. medical research council. Retrieved from https://www.mrc.ac.uk/documents/pdf/complex-interventions-guidance/

[CR6] Crane RS, Kuyken W, Hastings R, Rothwell N, Williams JMG (2010). Training teachers to deliver mindfulness-based interventions: Learning from the UK experience. Mindfulness.

[CR7] Crane RS, Kuyken W, Williams JMG, Hastings R, Cooper L, Fennell MJV (2012). Competence in teaching mindfulness-based courses: Concepts, development, and assessment. Mindfulness.

[CR8] Crane, R. S., Eames, C., Kuyken, W., Hastings, R. P., Williams, J. M. G., Bartley, T., …, Surawy, C. (2013). Development and validation of the Mindfulness-Based Interventions – Teaching Assessment Criteria (MBI:TAC). *Assessment, 20*(6), 681–688. 10.1177/1073191113490790 10.1177/1073191113490790 23794181

[CR9] Crane, R. S., Soulsby, J. G., Kuyken, W., Williams, J. M. G. and Eames, C. (2016). The bangor, exeter and oxford Mindfulness-Based Interventions: Teaching Assessment Criteria (MBI-TAC) for assessing the competence and adherence of mindfulness-based class-based teaching, Retrieved from https://www.bangor.ac.uk/mindfulness/documents/MBI-TACmanualsummaryaddendums05-16.pdf

[CR10] Crane RS, Brewer J, Feldman C, Kabat-Zinn J, Santorelli S, Williams JMG, Kuyken W (2017). What defines mindfulness-based programs? The warp and the weft. Psychological Medicine.

[CR11] Daubenmier, J., Moran, P. J., Kristeller, J., Acree, M., Bacchetti, P., Kemeny, M. E., …, Hecht, F. M. (2016). Effects of a mindfulness-based weight loss intervention in adults with obesity: A randomized clinical trial. *Obesity, 24*(4), 794–804. 10.1002/oby.21396 10.1002/oby.21396 PMC4898945 26955895

[CR12] Dimidjian S, Segal ZV (2015). Prospects for a clinical science of mindfulness-based interventions. American Psychologist.

[CR13] Dobkin PL, Hassed C (2016). Mindful medical practitioners: A guide for clinicians and educators.

[CR14] Dobkin PL, Hickman S, Monshat K (2013). Holding the heart of mindfulness-based stress reduction: Balancing fidelity and imagination when adapting MBSR. Mindfulness.

[CR15] European Association of Mindfulness based Approaches (EAMBA). (2017). Recommended ethical guidelines for mindfulness teachers. Retrieved from http://eamba.apps-1and1.net/about

[CR16] Evans, A., Crane, R. S., Cooper, L., Mardula, J., Wilks, J., Surawy, C., …, Kuyken, W. (2014). A framework for supervision for mindfulness-based teachers:A space for embodied mutual inquiry. *Mindfulness, 6*, 572–581. 10.1007/s12671-014-0292-4 10.1007/s12671-014-0292-4 PMC4432015 26000064

[CR17] Herschell AD (2010). Fidelity in the field: Developing infrastructure and fine-tuning measurement. Clinical psychology: Science and Practice.

[CR18] Hoffmann, T. C., Glasziou, P. P., Boutron, I., Milne, R., Perera, R., Moher, D., …, Michie, S. (2014). Better reporting of interventions: Template for intervention description and replication (TIDieR) checklist and guide. *British Medical Journal, 348*(g1687). 10.1136/bmj.g1687 10.1136/bmj.g1687 24609605

[CR19] Huijbers, M., Crane, R. S., Kuyken, W., Heijke, L., van den Hout, I., Donders, A. R. T., & Speckens, A. E. M. (2017). Mindfulness-based cognitive therapy; recurrent depression; intervention integrity; therapist competence; teacher competence. *Mindfulness*. 10.1007/s12671-016-0672-z 10.1007/s12671-016-0672-z PMC5506231 28757901

[CR20] Koo T, Li MA (2016). Guideline of selecting and reporting intraclass correlation coefficients for reliability research. Journal of Chiropractic Medicine.

[CR21] Kuyken, W., Nuthall, E., Byford, S., Crane, C., Dalgleish, T., Ford, T., …, Williams, J. M. G. (2017). The effectiveness and cost-effectiveness of a mindfulness training programme in schools compared with normal school provision (MYRIAD): Study protocol for a randomised controlled trial. *Trials, 18*, 194. 10.1186/s13063-017-1917-4 10.1186/s13063-017-1917-4 PMC5406917 28446223

[CR22] Ma SH, Teasdale JD (2004). Mindfulness-based cognitive therapy for depression: Replication and exploration of differential relapse prevention effects. Journal of Consulting and Clinical Psychology.

[CR23] Mackenzie CS, Poulin PA, Seidman-Carlson R (2006). A brief mindfulness-based stress reduction intervention for nurses and nurse aides. Applied Nursing Research.

[CR24] Marx R, Strauss C, Williamson C (2015). Mindfulness apprenticeship: A new model of NHS-based MBCT teacher training. Mindfulness.

[CR25] Mindfulness in School Project. (2017). The .b programme. Retrieved from https://mindfulnessinschools.org/.

[CR26] National Center for Complementary and Integrative Health (NCCIH). (2017). Framework for developing and testing mind and body interventions. Retrieved from https://nccih.nih.gov/grants/mindbody/framework

[CR27] Onken LS, Carroll KM, Shoham V, Cuthbert BN, Riddle M (2014). Reenvisioning clinical science: Unifying the discipline to improve the public health. Clinical Psychological Science.

[CR28] Peacock, J., Baer, R., Segal, Z. V., Crane, R. S., Kuyken, W. and Surawy, C. (2016). What is the role of retreats in Mindfulness-Based Cognitive Therapy for teachers? A dialogue on the perils, possibilities and ways forward. Retrieved from http://oxfordmindfulness.org/news/role-retreats-mbct-teachers/

[CR29] Perepletchikova F, Treat TA, Kazdin AE (2007). Treatment integrity in psychotherapy research: Analysis of the studies and examination of the associated factors. Journal of Consulting and Clinical Psychology.

[CR30] Prowse TP, Meadows GN, Enticott JC (2015). An exploratory study into the effectiveness of fidelity scales in the delivery of mindfulness-based cognitive therapy. Mindfulness.

[CR31] Ruijgrok-Lupton, P. E., Crane, R. S., and Dorjee, D. (2017). Impact of mindfulness-based teacher training on MBSR participant well-being outcomes and course satisfaction. *Mindfulness*, 1–12. 10.1007/s12671-017-0750-x 10.1007/s12671-017-0750-x PMC5770494 29387265

[CR32] Saito Y, Sozu T, Hamada C, Yoshimura I (2006). Effective number of subjects and number of raters for inter-rater reliability studies. Statistics in Medicine.

[CR33] Sanetti LMH, Kratochwill TR (2009). Toward developing a science of treatment integrity: Introduction to the special series. School Psychology Review.

[CR34] Santorelli, S. F., Kabat-Zinn, J., Blacker, M., Meleo-Meyer, F. and Koerbel, L. (2017). Mindfulness-based stress reduction (MBSR) authorized curriculum guide. Retrieved from http://www.umassmed.edu/cfm/training/mbsr-curriculum

[CR35] Schulz, K. F., Altman, D. G., Moher, D., for the CONSORT Group (2010). CONSORT 2010 Statement: updated guidelines for reporting parallel group randomised trials. *British Medical Journal, 340*, 698–792.

[CR36] Segal ZV, Teasdale JD, Williams JM, Gemar MC (2002). The mindfulness-based cognitive therapy adherence scale: Inter-rater reliability, adherence to protocol and treatment distinctiveness. Clinical Psychology and Psychotherapy.

[CR37] Segal ZV, Williams JMG, Teasdale JD (2013). Mindfulness-based cognitive therapy for depression.

[CR38] Segal, Z. V., Mark Williams, J. M. G., Teasdale, J. D., Crane, R. S., Dimidjian, S., Ma, H., …, Kuyken, W. (2016). Mindfulness-based cognitive therapy training pathway. Retrieved from http://oxfordmindfulness.org/wp-content/uploads/2016/10/MBCT-Training-Pathway-Final_Version1-0_07_Oct_2016-1.pdf

[CR39] Sharpless BA, Barber JP (2009). A conceptual and empirical review of the meaning, measurement, development, and teaching of intervention competence in clinical psychology. Clinical Psychology Review.

[CR40] Teasdale JD, Segal ZV, Williams JMG, Ridgeway VA, Soulsby JM, Lau MA (2000). Prevention of relapse/recurrence in major depression by mindfulness-based cognitive therapy. Journal of Consulting and Clinical Psychology.

[CR41] UK Network for Mindfulness-Based Teacher Training Organisations. (2016). Good practice guidance for mindfulness-based teachers. Retrieved from https://www.mindfulnessteachersuk.org.uk/

[CR42] van Aalderen JR, Breukers WJ, Reuzel RPB, Speckens AEM (2014). The role of the teacher in mindfulness-based approaches: A qualitative study. Mindfulness.

[CR43] Van Dam, N. T., van Vugt, M. K., Vago, D. R., Schmalzl, L., Saron, C. D., Olendzki, A., …, Meyer, D. E. (2017). Mind the hype: A critical evaluation and prescriptive agenda for research on mindfulness and meditation. *Perspectives on Psychological Science.*10.1177/1745691617709589 10.1177/1745691617709589 PMC5758421 29016274

[CR44] Vieten C, Astin J (2008). Effects of a mindfulness-based intervention during pregnancy on prenatal stress and mood: Results of a pilot study. Archives of Women's Mental Health.

[CR45] Weck F, Weigel M, Richtberg S, Stangier U (2011). Reliability of adherence and competence assessment in psychoeducational treatment influence of clinical experience. Journal of Nervous and Mental Disease.

